# Essential role of pre-existing humoral immunity in TLR9-mediated type I IFN response to recombinant AAV vectors in human whole blood

**DOI:** 10.3389/fimmu.2024.1354055

**Published:** 2024-06-28

**Authors:** Nada S. Alakhras, Christopher A. Moreland, Li Chin Wong, Priyam Raut, Sid Kamalakaran, Yi Wen, Robert W. Siegel, Laurent P. Malherbe

**Affiliations:** ^1^ Lilly Research Laboratories, Eli Lilly and Company, Indianapolis, IN, United States; ^2^ Prevail Therapeutics, a wholly owned subsidiary of Eli Lilly, New York, NY, United States

**Keywords:** AAV (Adeno-associated vector), plasmacytoid dendritic cells (pDCs), type I IFN, preexisting antibodies, TLR9

## Abstract

Recombinant adeno-associated virus (AAV) vectors have emerged as the preferred platform for gene therapy of rare human diseases. Despite the clinical promise, host immune responses to AAV vectors and transgene remain a major barrier to the development of successful AAV-based human gene therapies. Here, we assessed the human innate immune response to AAV9, the preferred serotype for AAV-mediated gene therapy of the CNS. We showed that AAV9 induced type I interferon (IFN) and IL-6 responses in human blood from healthy donors. This innate response was replicated with AAV6, required full viral particles, but was not observed in every donor. Depleting CpG motifs from the AAV transgene or inhibiting TLR9 signaling reduced type I IFN response to AAV9 in responding donors, highlighting the importance of TLR9-mediated DNA sensing for the innate response to AAV9. Remarkably, we further demonstrated that only seropositive donors with preexisting antibodies to AAV9 capsid mounted an innate immune response to AAV9 in human whole blood and that anti-AAV9 antibodies were necessary and sufficient to promote type I IFN release and plasmacytoid dendritic (pDC) cell activation in response to AAV9. Thus, our study reveals a previously unidentified requirement for AAV preexisting antibodies for TLR9-mediated type I IFN response to AAV9 in human blood.

## 1 Introduction

Recombinant adeno-associated virus (AAV) vectors are the leading platform for gene therapy for the treatment of patients with rare monogenic disorders. Despite the clinical promise, AAV immunogenicity has imposed a challenge to AAV clinical success (1, 2). Preexisting antibodies against the AAV capsids that can effectively block transduction in target cells are commonly found in humans (3, 4). Humoral and cellular immune responses against the AAV capsid that invariably developed after systemic infusion prevent successful vector re-administration (1). The cellular immune responses against the AAV capsid or transgene can also lead to the loss of transgene expression (5, 6). Therefore, a better understanding and mitigation of the risk factors inducing host immunity in AAV-mediated gene therapy is key to achieving safe and efficient gene therapy.

Preclinical models have highlighted the essential role played by the innate immune cells in shaping the adaptive immune response to AAV. Recognition of AAV is thought to be mediated by the pattern recognition receptors TLR2 and TLR9. TLR9 is located in the endosomal compartment and recognizes unmethylated CpG (cytosine-phosphate-guanine dideoxynucleotide) DNA motifs (7) while TLR2 is located on the cell surface and recognizes glycoprotein and lipoprotein (8). In murine models, TLR9 sensing of AAV in plasmacytoid dendritic cells (pDCs) and the resulting type I IFN response are critical for the induction of the cellular and humoral immune response to AAV (9, 10). Consistent with an essential role of TLR9 signaling in human, depleting CpG motifs from the transgene has been shown to mitigate AAV-immune response and enhance transgene expression in clinic (11). Surprisingly, however, several recent studies have reported that AAV failed to activate human pDC and the release of type I IFN *in vitro* (12, 13), suggesting that human myeloid DC and IL-6 release played a more preponderant role in the innate immune response to AAV in human.

In this study, we assessed the innate immune responses to AAV9, the preferred serotype for AAV-mediated gene therapy of the CNS. We found that AAV9, like AAV6, triggered type I IFN and IL-6 responses in human whole blood. We demonstrated that type I IFN production is mediated by TLR9 signaling. Importantly, we uncovered a requirement for the preexisting antibodies against AAV9 for TLR9-mediated pDC activation and type I IFN production in response to AAV9. These results reveal previously unrecognized mechanisms regulating TLR9 activation by AAV and could provide a strategy to mitigate AAV immunogenicity in clinical studies.

## 2 Materials and methods

### 2.1 AAV vectors

Single-stranded AAV9-GFP and AAV6-GFP viral vectors were produced through a dual-infection process where two recombinant baculoviruses (rBV) are used to infect *Spodoptera frugiperda* (Sf9) insect cells. The two baculoviruses used include one rBV containing the viral genes for AAV9 or AAV6 capsid formation and another rBV that contains the GFP gene with a CBA promoter. Upstream material is purified by chromatography and tangential flow filtration to yield a high purity final vector.

Single-stranded AAV9-HEK vectors carrying a human transgene were produced at Nationwide Children’s (Columbus, OH) by triple transfection of HEK293 cells, purified and concentrated by cesium chloride gradient purification. QC testing included purity check by SDS-PAGE/Silver stain, genomic titer by qPCR. The vectors were stored at −80°C in storage buffer composed of 20 mM Tris (pH 8.0), 200 mM NaCl, 1 mM MgCl2, and 0.001% Poloxamer 188.

Empty AAV9 vector (PR001E-22–351) and custom-prepared single-stranded AAV9 vectors carrying a human transgene with or without CpG depletion were produced in Sf9 insect cells by Virovek using the same manufacturing process. The vectors were purified through 2 rounds of cesium chloride ultracentrifugations. The cesium chloride was removed through buffer exchange with 2 PD-10 desalting columns in 1 x PBS + 0.001% pluronic F-68. All the vectors used were single-stranded vectors.

### 2.2 AAV9 antibodies

Naturally occurring preexisting AAV9 antibodies were purified from AAV9 positive sera using an affinity purification assay. Briefly, AAV9 seropositive sera were pooled and incubated with Affinity-Gel10 resin overnight at 4°C. Post incubation, the resin was packed to a column and washed with 10x resin volume of PBS, 20x resin volume 10 mM pH 7.5 Tris-HCl + 0.5 M NaCl, and 10x resin volume of 10 mM pH 7.5 Tris-HCl, respectively. Anti-AAV9 antibodies were eluted with 100 mM pH 2.5 glycine and immediately neutralized with 1/10 volume of 1 M pH 8 Tris-HCl. Fractions containing antibodies were concentrated and dialyzed against pH 7.4 PBS.

Recombinant human chimeric monoclonal antibody was derived from murine monoclonal antibody immunized with AAV9 viral particles. The variable region sequence was cloned into expression vectors containing human IGKC and IGHG1 constant region sequences and transiently transfected into CHO cells. Culture supernatants were harvested after 7 days, and antibody was purified using Mab SureSelect resin. Anti-AAV9 antibodies were eluted with 100 mM pH 2.5 glycine and immediately neutralized with 1/10 volume of 1 M pH 8 Tris-HCl. Fractions containing antibodies were concentrated and dialyzed against pH 7.4 PBS.

### 2.3 Human blood samples

Human blood samples were obtained from healthy donors after approved consent by the ethical practice of the Lilly Research Biological Donation Program (RBD) committee.

### 2.4 Whole blood assay

Whole blood samples from healthy cohorts were collected in Na heparin tubes and used within two hours after donation (14). Blood was added at 225 μl in two biological replicates into 96-well plates. AAV9 or AAV6 vectors were added to the blood samples at three titers: 1, 10, and 30 E10 vg with a total volume of 250 μl per well. Additionally, whole blood was treated with PBS buffer and used as an unstimulated negative control. Blood samples were also stimulated with 5μg/ml of a Class C CpG oligonucleotide (CpGC) (InvivoGen, ODN2395, Cat# tlrl-2395) as a positive control for TLR9 activation. Following 24-hour incubation at 37°C, 5% CO_2_ plates were centrifuged at 1,000 g, and blood plasma supernatants were collected.

### 2.5 Cytokine multiplex assays

Pro-inflammatory cytokine concentration in plasma supernatants were detected using an MSD multiplex human pro-inflammatory panel assay (Meso Scale Discovery, Cat# K15049D-2) following manufacturer instructions. Duplicate plasma supernatants derived from the whole blood assay were analyzed for the following cytokines: IFN-γ, IL-1β, IL-2, IL-4, IL-6, IL-8, IL-10, IL-12p70, IL-13, and TNF-α.

### 2.6 Human Interferon Alpha ELISA

The concentrations of IFN-α in the plasma were determined using an IFN-α multi-subtype ELISA (Cat#41110–2, PBL) following manufacturer instructions. Concentration is reported in (pg/ml). The concentration values below IFN-α assay detection limits were assigned a value of (1).

### 2.7 TLR9 inhibition

Blood samples were incubated with TLR9 inhibitor E6446 (Selleckchem.com, Cat#S0716) at three concentrations: 0.2, 2, and 20 µM for 60 minutes. Then, the samples were stimulated with AAV9 at 10 E10 vg for 24 hours.

### 2.8 Total antibody (TAb) assay

Levels of AAV9 preexisting antibodies in human plasma were measured using an affinity capture and elution (ACE) TAb assay (15). Briefly, AAV9-GFP was coated in a Nunc Maxisorp 96-well plate. Human serum was diluted 1:10 in Tris-buffered saline and added to the plate, followed by overnight incubation at 4°C to capture anti-AAV9 antibodies. The plate was washed, and captured antibodies were eluted with 300 mM acetic acid and transferred to an MSD (MesoScale Discovery) plate containing 1 M pH 9.5 Tris-HCl neutralization buffer. The MSD plate was incubated for two hours at room temperature to allow the immobilization of anti-AAV9 antibodies. The MSD plate was washed, and ruthenium labeled AAV9-GFP was added. After a final wash, a 2X MSD read buffer was added, and signals were collected in an MSD Quick Plex SQ 120 reader.

### 2.9 Spiking anti-AAV9 antibodies

Blood samples were obtained from AA9 seronegative donors, incubated with purified 50 ug of polyclonal anti-AAV9 derived from seropositive sera for 20 minutes to 1 hour, and then stimulated with AAV9 at 10 E10 vg or PBS control for 24 hours.

### 2.10 Flow cytometry

Blood cells from AAV9 treated samples were surface stained with fluorochrome-conjugated monoclonal antibodies specific for human HLA-DR (clone L243, BV785 Cat# 307642), CD89 (clone A59, BV510), CD88 (clone S5/1), CD3 (clone SK7, AF700 Cat# 302226), CD19 (clone 4G7/HIB19, AF700 Cat# 302226), CD56 (clone HCD56, AF700 Cat# 318316), CD66b (clone G10F5, AF700 Cat#305114), (CD11c (clone B-Ly6, BV650 Cat# 563403), CD123 (clone 6H6, BV711 Cat# 306030), CD83 (clone HB15, FITC Cat#305306), and CD86 (clone IT2.2, PE Cat#3054050) all from Biolegend or BD Bioscience. Red blood cells were lysed with lysis buffer (BD PharmLyse) for 10 minutes and then washed two times with PBS. Cells were incubated with fixable viability stain 450 (BD Biosciences, Cat# 562247, 1:1000 dilution) for 15 minutes. Cells were washed and fixed with 2% of paraformaldehyde. Cells were analyzed using Cytek Aurora (Cytek), and the data were analyzed with FlowJo software v10.9.0 (BD).

### 2.11 Statistics

All data were analyzed using GraphPad Prism 9.2 (Software) or JMP-16. Statistical significance was determined by paired two-tailed t-test or one-way analysis of variance (ANOVA) with multiple comparison test. The statistical tests for each figure are stated in the corresponding figure legend. Data are presented as mean ± standard error of the mean (SEM).

## 3 Results

### 3.1 AAV9 and AAV6 serotypes induce type I IFN response in human whole blood

**Figure 1:**
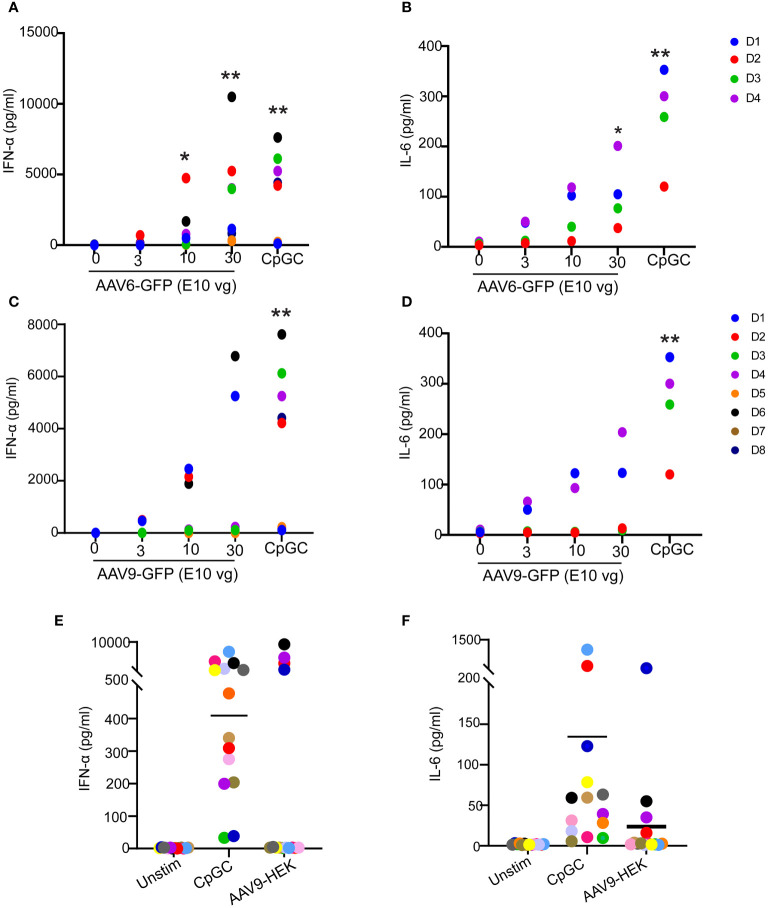
Innate immune responses to AAV serotypes in human blood. **(A–D)** Whole blood from healthy human donors was stimulated with AAV6-GFP or AAV9-GFP at 0,3,10, and 30 E10 vg or TLR9 agonist CpGC. Scatter plots of secreted IFN-α and IL-6 in response to AAV6-GFP **(A, B)** or AAV9-GFP **(C, D).** Data in **(A, C)** were combined from two independent experiments (n=8 donors). Data in **(B, D)** represent one independent experiment with (n=4). **(E, F)** Whole blood from healthy human donors was stimulated with mammalian cell-derived AAV9-HEK carrying a human transgene at 10 E10 vg or TLR9 agonist CpGC. Scatter plots of secreted IFN-α **(E)** and IL-6 **(F)** in response to AAV9-HEK. *P*-value was determined based on a one-way ANOVA nonparametric multiple comparison test with Dunn method, *P <0.05, and ^**^ *P <*0.01. “Unstim” indicates unstimulated.

To characterize the innate immune response to AAV, we stimulated human blood samples from healthy donors with different doses of AAV6 or AAV9 vectors carrying the GFP transgene and analyzed the secreted levels of proinflammatory cytokines. We detected robust induction of IFN-α (**Figures 1A, C**) and IL-6 (**Figures 1B, D**) responses to both AAV6-GFP and AAV9-GFP. While all donors responded to AAV6-GFP at the highest titers tested, only half of the donors responded to AAV9-GFP (**Figures 1A–D**). Remarkably, all donors produced type I IFN and IL-6 as well as other inflammatory cytokines (TNF, IL-1β, IL-8) in response to the TLR9 agonist CpGC (**Figures 1A–D**; **Supplementary Figure 1**; **Supplementary Table 1**). This pattern of cytokine response was not unique to insect cells-derived AAV vectors carrying the GFP transgene and was also reproduced with AAV9 vectors produced by mammalian HEK cells and carrying a human transgene (**Figures 1E, F**). These observations suggest that AAVs can induce type I IFN and IL-6 release in human whole blood, but this innate response is donor-dependent.

### 3.2 CpG motifs recognition mediates innate cytokine secretion in human blood.

**Figure 2:**
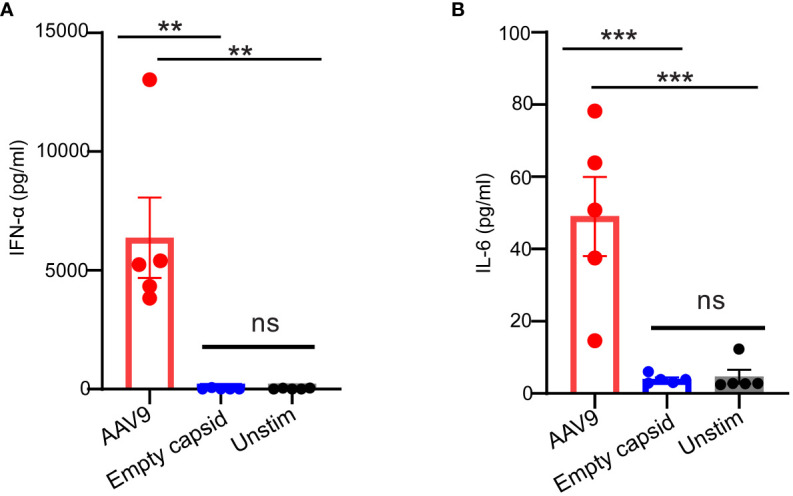
AAV transgene but not the empty capsid mediates cytokine response in human whole blood. Whole blood samples from healthy human donors identified previously as responders to AAV9 vector were stimulated with empty AAV9 vectors or AAV9 vectors carrying a human transgene at 10 E10 vg or kept unstimulated. Scatter plots of secreted **(A)** IFN-α **(B)** or IL-6 concentrations. Data represent mean ± SEM, (n= 5). *P*-value was determined based on one-way ANOVA with Tukey *post-hoc* t-test. ^**^ *P <*0.01, ^***^ *P <*0.001. “Unstim” indicates unstimulated. ns indicates not significant.

A recent study suggested that full and empty viral particles were equally potent in inducing cytokine secretion in human blood (12). To understand the relative contribution of the capsid and transgene to the innate immune response to AAV in our human whole blood assay, we compared the innate response to full and empty AAV9 viral particles produced from insect cells using the same manufacturing process. While the full AAV9 capsid elicited the secretion of IFN-α and IL-6, the empty AAV9 capsid failed to trigger any cytokine secretion in these donors (**Figures 2A, B**) suggesting that the transgene plays a predominant role in the innate response to AAV in human whole blood.

**Figure 3:**
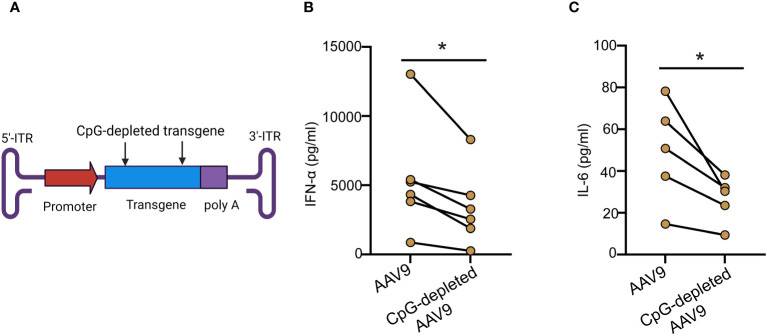
Cytokine responses are reduced with CpG depletion. **(A)** Schematic illustration of the AAV9 vector showing the CpG-depleted transgene site. **(B, C)** Whole blood from healthy human donors was treated with AAV9 vectors carrying a human transgene with or without CpG depletion for 24 hours. **(B)** Scatter plot of the IFN-α **(C)** and IL-6 concentrations in the plasma. Data represent mean ± SEM and represent two independent experiments (n=6 or 5). *P*-value was determined based on a two-tailed paired t-test. ^*^ *P <*0.05.

Decreasing the amount of CpG motifs in the transgene sequence has been associated with enhanced AAV-mediated gene expression in clinic (11). To assess the impact of CpG content on the innate response to AAV9, we prepared a CpG-depleted AAV9 vector where CpG motifs were depleted from the codon-optimized protein coding portion (CDS) of a human transgene but not the promoter and inverted terminal repeat (ITR) sequences (**Figure 3A**). Stimulation of the human blood cells with CpG-depleted AAV9 significantly reduced IFN-α and IL-6 levels compared to the stimulation with the AAV9 vector carrying the codon-optimized human transgene but without any depletion of the CpG motifs (**Figures 3B, C**). Overall, these results demonstrate that the innate immune response to AAV9 in human whole blood is dominated by the recognition of the unmethylated CpG sites from the AAV expression cassette.

### 3.3 TLR9 mediates Type I IFN response to AAV9 and AAV6 in human blood

**Figure 4:**
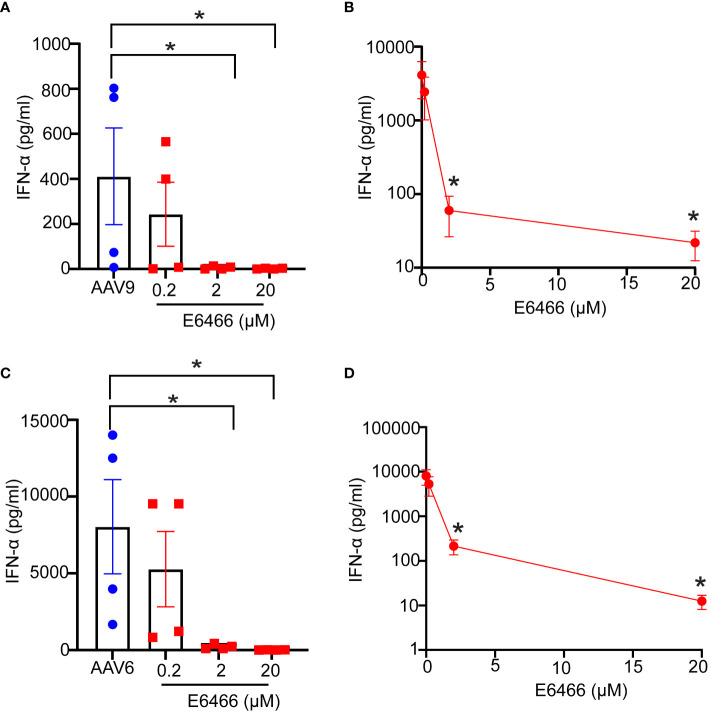
Inhibition of TLR9 suppressed Type IFN response to AAV. Whole blood samples from healthy human donors identified previously as responders to AAV9 or AAV6 vectors were incubated with E6466 inhibitor at three concentrations 0.2, 2, or 10 µM for one hour prior to stimulation with AAV9-GFP or AAV6-GFP at 10 E10 vg for 24 hours. **(A)** Scatter plots and **(B)** line plot of concentration-dependent E6466 inhibition of IFN-α response AAV9-GFP. **(C)** Scatter plots and **(D)** line plot of concentration-dependent E6466 inhibition of IFN-α response to AAV6-GFP. Data represent mean ± SEM and representative of two independent experiments (n=4 per experiment). *P*-value was determined based one-way ANOVA with nonparametric multiple comparison Wilcoxon method, ^*^ *P <*0.05.

Previous reports have demonstrated a critical role in mice for TLR9-mediated type I IFN secretion due to AAV (10). Whether AAV mediates TLR9 activation and type I IFN response in humans remains however controversial (12, 16). To examine the importance of TLR9 signaling in human blood response to AAV, we used E6446, an antagonist of nucleic acid sensing-TLRs (17). E6446 specifically inhibits TLR9 activation in mice and humans (18–20). We found that stimulating the blood samples with AAV9-GFP (**Figures 4A, B**) or AAV6-GFP (**Figures 4C, D**) in the presence of E6446 diminished the secretion of IFN-α levels in human blood in a concentration-dependent manner (**Figures 4A, B**). Similar dose-dependent inhibition was observed for human blood samples stimulated with CpGC (**Supplementary Figure 2**). Thus, these results demonstrate that TLR9 mediates type I IFN response to AAV in humans.

### 3.4 Preexisting antibodies are required for TLR9-mediated IFN response to AAV9

**Figure 5:**
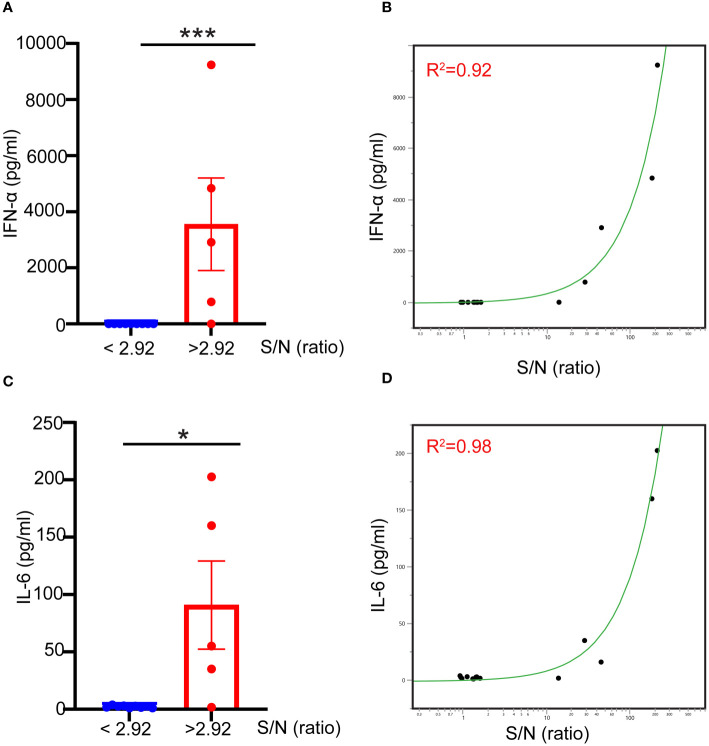
Naturally occurring preexisting antibody levels in human blood correlate with type I IFN response to AAV. Blood samples were obtained from healthy donors. The samples were either stimulated with mammalian cell-derived AAV9-HEK carrying a human transgene at 10 E10 vg for 24 hours or centrifuged to obtain naïve blood-derived plasma. **(A)** Scatter plot showing the correlation of the levels of naturally occurring preexisting antibodies measured with TAb assay in donors’ plasma to the IFN-α responses to AAV9. **(B)** Linear fit graph showing the correlation between the levels of naturally occurring preexisting antibodies against AAV9 in donors’ plasma to IFN-α responses. **(C)** Scatter plot showing the levels of naturally occurring preexisting antibodies in donors’ sera to IL-6 responses to AAV9. **(D)** Linear fit graph showing the correlation between the levels of naturally occurring preexisting antibodies in donors’ sera to IL-6 responses. *P*-value was determined based on a two-tailed paired t-test. ^*^ *P <*0.05, ^***^ *P <*0.001. The S/N ratio indicates the ratio of the assay signal to noise in Tab assay, S/N level ≥2.92 indicates a seronegative donor whereas S/N levels ≤ 2.92 indicates a seropositive donor. All values below IFN-α assay detection limits were assigned a value of (1).

Naturally occurring preexisting antibodies against AAV have been shown to improve AAV uptake by blood phagocytes and enhance the proinflammatory cytokine responses in human blood (12). To assess the role played by naturally occurring antibodies against AAV9 to the innate immune response to AAV9 in the whole blood assay, we divided blood samples into seronegative (signal-to-noise ratio (S/N) ≤2.92), or seropositive (S/N > 2.92) using an assay described previously (15) and measured their capacity to mount an innate immune response to AAV9. We found that seronegative donors exhibited no IFN-α or IL-6 responses when stimulated with AAV9 for 24 hours (**Figure 5**). In contrast, seropositive donors (with the exception of one donor with the lowest S/N ratio) exhibited potent and robust IFN-α and IL-6 responses, and the magnitude of the cytokine response to AAV9 correlated with the level of preexisting antibody (R^2^ = 0.92 and R^2^ = 0.98 for IFN-α and IL-6, respectively). Thus, these findings demonstrate that the presence of a preexisting antibody is necessary for the AAV9 induction of type I IFN in human whole blood.

**Figure 6:**
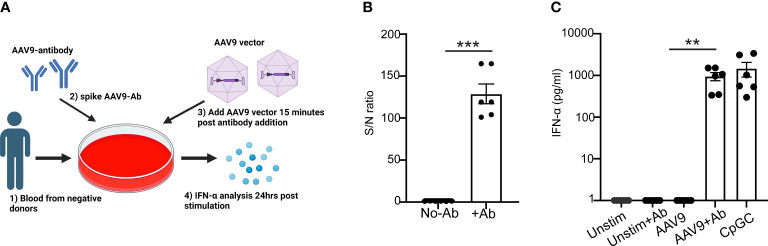
Anti-AAV9 antibodies are sufficient to promote type I IFN response to AAV9 in human whole blood. **(A)** Schematic illustration of the anti-AAV9 spiking to AAV9-negative donors prior to stimulating the blood with mammalian cell-derived AAV9-HEK carrying a human transgene at 10 E10 vg. Following 24 hours of stimulation, plasma samples were collected and the levels of secreted IFNα were evaluated. **(B)** Scatter plot comparing the level of anti-AAV9 in naive plasma samples derived from whole blood of AAV9-negative donors before and after spiking the anti-AAV9 levels measured with Tab assay. **(C)** Scatter plot of the IFN-α concentrations at the indicated conditions. Data represent mean ± SEM and represent two independent experiments (n=6). The *P*-value was determined based on a paired t-test. ^**^ *P <*0.01, ^***^ *P <*0.001. Unstim indicates unstimulated. All values below IFN-α assay detection limits were assigned a value of (1).

To determine whether anti-AAV9 antibodies are sufficient to trigger an innate immune response to AAV9, purified polyclonal anti-AAV9 antibodies derived from the sera of seropositive donors were spiked to the blood samples from seronegative donors before exposure to AAV9 (**Figures 6A, B**). Spiking the blood samples of seronegative donors with purified polyclonal anti-AAV9 antibodies was sufficient to restore the capacity of these blood samples to mount a robust type I IFN to AAV9 (**Figure 6C**). Collectively, these results demonstrate that the essential role of anti-AAV9 antibodies for the innate immune response to AAV9 in human whole blood.

### 3.5 Plasmacytoid DC (pDC) activation by AAV9 in human whole blood requires preexisting antibodies

**Figure 7:**
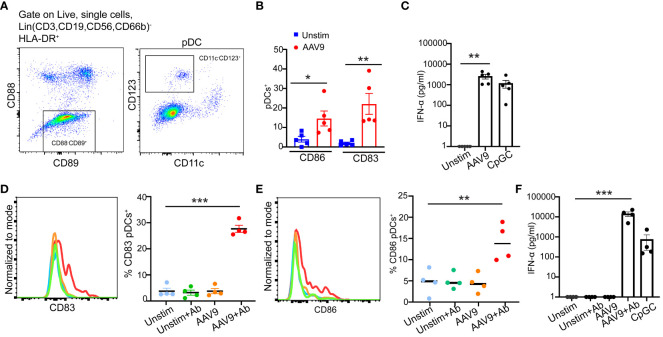
Preexisting antibodies against AAV9 promote pDC activation and type I IFN secretion. **(A)** Gating strategy to identify pDCs in human blood. **(B)** Human blood from seropositive donors were stimulated for 24 hours with mammalian cell-derived AAV9-HEK carrying a human transgene at 10 E10 vg or kept unstimulated. Frequencies of CD83 or CD86 expressions on the surface of pDCs. **(C)** Scatter plot of IFN-α concentrations at the indicated conditions **(D)** Human blood cells from seronegative donors were stimulated with AAV9 for 24 hours or kept unstimulated (Unstim) in the presence or absence of spiked anti-AAV9 antibodies. Representative histogram and scatter plot of the frequencies of CD83 expressions on pDCs indicated conditions. **(E)** Representative histogram and scatter plot of the frequencies of CD83 and CD86 expressions on pDCs at the indicated conditions. **(F)** Scatter plot of IFN-α concentrations at the indicated conditions. Data represent mean ± SEM and two independent experiments (n=5 or 4). The *P*-value was determined based on one-way ANOVA with Tukey multiple comparison test. ^*^ *P <*0.05, ^**^ *P <*0.01, ^***^ *P <*0.001. Unstim indicates unstimulated. All values below IFN-α assay detection limits were assigned a value of (1).

pDCs play a critical role in sensing and recognizing viruses including AAV through TLR9 on the surface of the endosomal compartment (21). Previously, studies demonstrated that human pDCs upregulate the expression of costimulatory markers CD86, CD83, and CD40 upon activation (22, 23). Thus, we sought to examine pDC activation by AAV9 in human blood. We first stimulated blood samples from seropositive donors with AAV9 for 24 hours. To accurately identify pDCs in the blood, we used a gating strategy (24–26) and identified pDCs as CD11c^-^CD123^+^ (**Figure 7A**). Stimulation with AAV9 significantly increased the expression of CD86 and CD83 on pDCs in seropositive donors stimulated with AAV9 relative to unstimulated cells (**Figure 7B**) which coincided with IFN-α secretion (**Figure 7C**).

To determine if the presence of anti-AAV9 antibody is sufficient to promote pDC activation by AAV9 in human blood, we spiked blood samples from seronegative donors with monoclonal anti-AAV9 antibodies. pDCs from seronegative donors only increased their expression of CD86 and CD83 in response to AAV9 when the blood samples were spiked with anti-AAV9 antibodies. (**Figures 7D, E**). In line with pDC activation and previous results with purified polyclonal antibodies, spiking monoclonal anti-AAV9 antibodies in the blood samples of seronegative donors was sufficient to trigger a potent IFN-α secretion in response to AAV9 (**Figure 7F**). Overall, our results demonstrated that pDC activation by AAV9 in human whole blood requires the presence of anti-AAV9 antibodies.

## 4 Discussion

Although recombinant AAV for gene transfer has been successfully used to treat rare monogenic diseases, innate immune activation and acute toxicities have been reported with AAV treatment in clinical studies (27, 28). Here, we examined the innate immune response to AAV and demonstrated that AAV activates human pDCs and promotes TLR9-mediated type I IFN secretion. Importantly, we demonstrate a requirement for the preexisting antibodies against AAV9 for TLR9-mediated pDCs activation and type I IFN secretion in human blood.

In our study, we detected a robust type I IFN secretion to two different AAV serotypes. Type I IFN is a critical cytokine produced as a defense response to viruses. In contrast, previously published report failed to detect type I IFN to AAV stereotypes in human blood (12). One explanation for this discrepancy in AAV responses in human blood could be the choice of anticoagulant for blood collection. It has been shown that the choice of anticoagulants can not only impact complement activation (29) but also leukocyte activation, and cytokine and chemokine production in response to TLR agonists in human whole blood (29–32). Although the precise mechanisms involved are not fully understood, anticoagulants can impact cell-cell interactions in human blood and thereby affect blood cell activation and cytokine production. For example, citrated blood that was used in the studies of Smith et al. to study innate response to AAV is known to interfere with Ca^2+^-dependent interactions between platelets and monocytes and reduced platelet-leukocyte interactions compared with heparinized blood that was used in our study (33). Overall, the methods of collecting and stimulating the blood with AAV could have influenced the discrepancy in detecting type I IFN to AAV in whole blood.

In agreement with previously published data in mice, we detected pDC activation to AAV9. We showed increased expression of the activation markers CD86 and CD83 on pDCs, which correlated with type I IFN secretion. Findings in mice demonstrated that AAV activates pDC through TLR9-MyD88 pathway to secrete type I IFNs (10). In human blood, pDC and B cells express the highest levels of TLR9 expression, and they are the most sensitive cells to CpG DNA (34). Our findings showed that the inhibition of TLR9 diminished type I IFN secretion to AAV viral DNA in human blood indicating that this response is mediated by TLR9. Although B cells in human blood express TLR9, pDCs are the main producer of type I IFNs through TLR9 (9, 21, 35). Thus, our findings demonstrate that, similar to what was observed in mouse, AAV activates pDCs in human blood.

Our study demonstrates the requirement of the preexisting antibodies for pDCs activation by AAV9. Smith et al. previously reported the critical role played by preexisting antibodies to AAV for the response of myeloid DC in human whole blood (12). Our study further extends the idea that preexisting humoral immunity to AAV enhanced the innate response to AAV by impacting the response of pDC. AAV serotypes bind glycans on the surface of the cells as their primary receptor and the presence of co-receptors on cell surface facilitates their entry to the cells (36). AAV9 achieves efficient transduction in muscle cells, hepatocytes, and neurons (37). One explanation of our findings is that pDCs in human blood lack AAV9 co-receptor and therefore poorly internalize AAV9 (12). Preexisting antibodies could form an immune complex with AAV9 capsid facilitating the uptake of AAV9 by pDCs via their Fc gamma receptors and the recognition of the CpG motifs in the transgene by TLR9 in the endosomal compartment. Such Fc-mediated uptake has been demonstrated for various viruses including influenza, HIV, and SARS-CoV (38–40). More generally, DNA viruses and related vectors that contain CpG motifs may not stimulate TLR9 in human pDC as readily as synthetic TLR9 agonists such as CpGC. For example, human pDCs have been shown to produce high amounts of type I IFN when incubated with live CMV-infected fibroblasts, but not with free CMV (35).

In conclusion, our results confirm the importance played by TLR9 recognition by pDC in the human innate immune response to AAV and suggest that the preexisting humoral immunity to AAV vectors may be an important determinant in the immune safety of AAV-based gene therapy by promoting TLR9-mediated type I IFN response to recombinant AAV vectors.

## Data availability statement

The raw data supporting the conclusions of this article will be made available by the authors, without undue reservation.

## Ethics statement

The studies involving humans were approved by the Lilly Research Biological Donation Program (RBD) committee. The studies were conducted in accordance with the local legislation and institutional requirements. The human samples used in this study were acquired from All donors provided their written agreement for using their blood for research purposes. This study used deidentified blood samples from healthy donors after approved consent by the ethical practice of the Lilly Research Biological Donation Program (RBD) committee. Written informed consent for participation was not required from the participants or the participants’ legal guardians/next of kin in accordance with the national legislation and institutional requirements.

## Author contributions

NA: Conceptualization, Data curation, Formal analysis, Investigation, Methodology, Software, Visualization, Writing – original draft, Writing – review & editing. CM: Investigation, Writing – review & editing. LW: Project administration, Resources, Writing – review & editing. PR: Conceptualization, Methodology, Writing – review & editing. SK: Conceptualization, Methodology, Writing – review & editing. YW: Conceptualization, Formal analysis, Investigation, Methodology, Writing – review & editing. RS: Conceptualization, Methodology, Writing – review & editing. LM: Conceptualization, Formal analysis, Project administration, Resources, Supervision, Writing – review & editing.
