# Supplementary material for: Essential role of pre-existing humoral immunity in TLR9-mediated type I IFN response to recombinant AAV vectors in human whole blood
Source: Front Immunol. 2024 Jun 28;15:1354055. doi: 10.3389/fimmu.2024.1354055 (PMC11240241; doi:10.3389/fimmu.2024.1354055)
Supplement: Supplementary file 1 [file DataSheet_1.docx]

Supplementary Material

Essential role of preexisting humoral immunity in TLR9-mediated type I IFN response to recombinant AAV vectors in human whole blood

**Nada S. Alakhras^1^, Christopher A. Moreland^1^, Li Chin Wong^2^, Priyam Raut^2^, Sid Kamalakaran^2^, Yi Wen^1^, Robert W. Siegel^1^, Laurent P. Malherbe^1^**

**** Correspondence:** Laurent Malherbe: [malherbe_laurent@lilly.com](mailto:malherbe_laurent@lilly.com)

# Supplementary Data


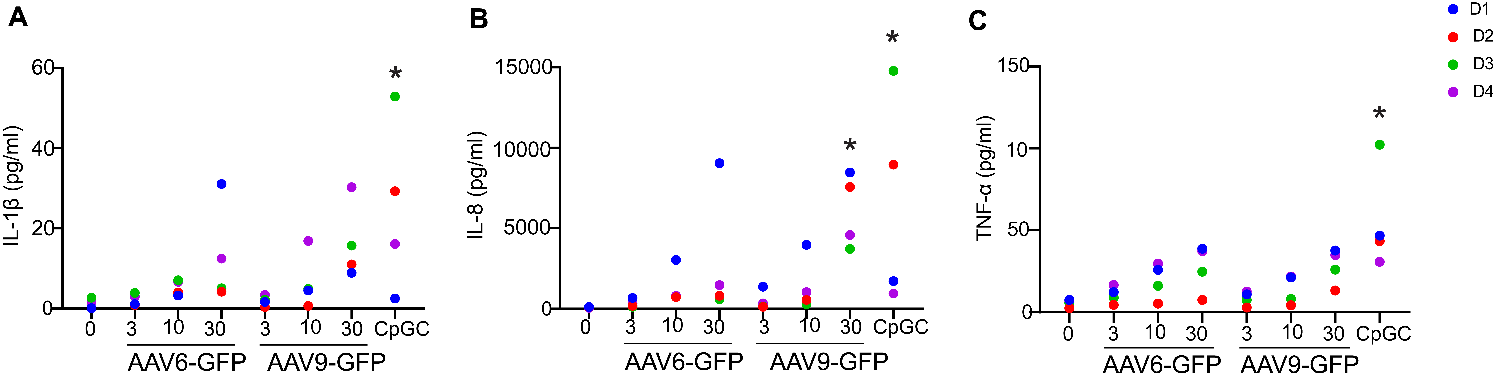


**Supplementary Figure 1.** Innate immune responses to AAV serotypes in human blood. Whole blood from healthy human donors was stimulated with insect-derived AAV6-GFP or AAV9-GFP at 0,3,10, and 30 E10 vg or TLR9 agonist CpGC. Scatter plots of secreted proinflammatory cytokines to AAV6-GFP and AAV9-GFP **(A)** IL-1β, **(B)** IL-8, and **(C)** TNF-α. Data represent mean ± SEM and represent one independent experiment with (n=4). *P*-value was determined based on a one-way ANOVA nonparametric multiple comparison test with the Dunn method, *P <0.05.


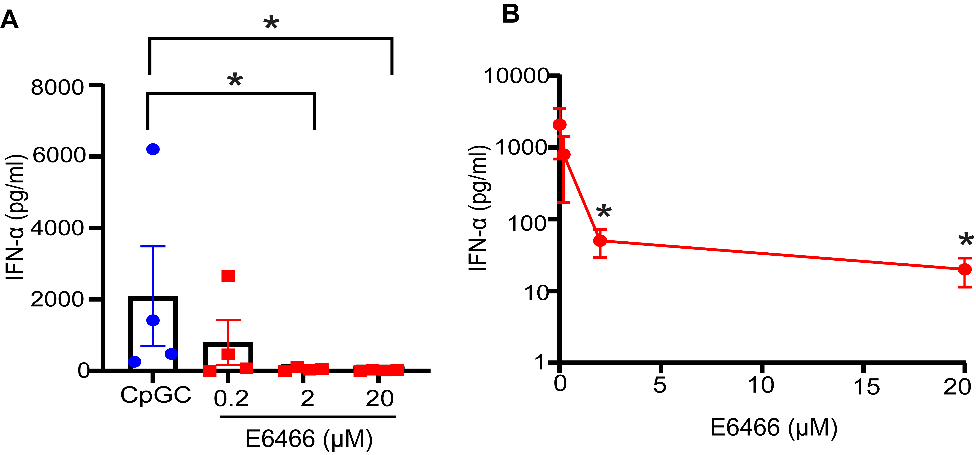


**Supplementary Figure 2.** Inhibition of TLR9 suppressed Type IFN response to AAV. Whole blood samples from healthy human donors were incubated with E6466 inhibitor at three concentrations 0.2, 2, or 10 µM for one hour prior to stimulation with CpGC at 5µg/ml for 24 hours. (A) Scatter plots and (B) line plot of concentration-dependent E6466 inhibition of IFN-α response to CpG-C. Data represent mean ± SEM and representative of two independent experiments (n=4 per experiment). *P*-value was determined based one-way ANOVA nonparametric multiple comparison test with the Wilcoxon method, **P* <0.05.
